# Fault material heterogeneity controls deep interplate earthquakes

**DOI:** 10.1126/sciadv.adr9353

**Published:** 2025-02-26

**Authors:** Yihe Huang, Satoshi Ide, Aitaro Kato, Keisuke Yoshida, Chengxin Jiang, Peng Zhai

**Affiliations:** ^1^ Department of Earth and Environment Sciences, University of Michigan, Ann Arbor, MI 48104, USA.; ^2^ Department of Earth and Planetary Science, University of Tokyo, Tokyo 113-8654, Japan.; ^3^ Earthquake Research Institute, University of Tokyo, Tokyo 113-0032, Japan.; ^4^ Graduate School of Science, Tohoku University, Sendai 980-8577, Japan.; ^5^ Research School of Earth Sciences, Australian National University, Canberra 0200, Australia.

## Abstract

Earthquakes may seem random, but are often concentrated in some localized areas. Thus, they are likely controlled by fault materials and stress heterogeneity, which are little understood. Here, we provide high-resolution observations of fault material and stress heterogeneity in the Japan subduction zone through an integration of material and source imaging with numerical simulations. Our results present evidence for localized, anisotropic structures with a near-zero Poisson’s ratio in the medium surrounding 1 to 2 kilometer–sized earthquake clusters, suggesting that the fault medium is damaged, foliated, and enriched with fluid. Such localized structures may cause stress perturbations on faults that in turn favor the frequent occurrence of deep interplate earthquakes at depths of 60 to 70 kilometers. Therefore, identifying the distribution and properties of fault material heterogeneity is important for more informed assessment of earthquake hazards.

## Teaser

Heterogeneous kilometer-scale structures on the subduction zone plate interface control where deep interplate earthquakes occur.

## INTRODUCTION

It has long been thought that fault stress heterogeneity plays a central role in the generation of earthquakes and may facilitate or stop earthquake rupture propagation (*1*). Material heterogeneity, on the other hand, has been considered to play a secondary role, although its effects on source characteristics of earthquakes have been recognized in laboratory experiments (*2*–*4*) and observations (*5*–*7*). Recent numerical models show that material heterogeneity, such as fault damage zones manifested as volumes of fractured rocks surrounding faults (*8*, *9*), can give rise to stress modulation on faults and is directly responsible for the temporal variation of earthquake magnitudes and recurrence intervals (*10*). The fractures inside damage zones may act as conduits for fluid propagation when they are well connected (*9*, *11*), leading to cyclic changes in pore pressure along faults that promote the alternation between earthquakes and slow-slip transients (*12*). These numerical results suggest that the effects of material and stress heterogeneity are inevitably intertwined and contribute together to the generation of earthquakes. Thus, understanding the distribution and properties of fault material heterogeneity provides vital information about the possible earthquake mechanisms.

**Fig. 1.**
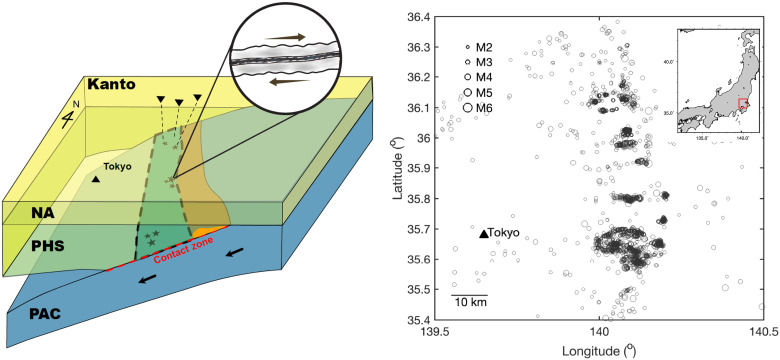
Schematic representation of the subduction zone underneath the Kanto region (left) and spatial distribution of M2 to M6 earthquakes at depths of 60 to 70 km between 2004 and 2020 based on double-difference relocation results (right). The schematic figure, modified from Fig. 2 in Uchida *et al.* (*13*) and Fig. 5 in Ishise *et al.* (*15*), shows the seismic stations (triangles) and the localizations of deep interplate earthquakes (stars) between the PHS and PAC. The green region bounded by dashed lines highlights the seismically active area between the PHS and PAC at 60 to 70 km, while the orange area above the green region shows the serpentinized mantle of the PHS inferred by tomography models (*21*). The contact area between the PHS and PAC is highlighted by the red dashed line. The inset figures demonstrate the foliated shear zone inferred by our study (left) and a map of Japanese Islands with the study region highlighted in red (right).

The frequent and spatially localized occurrence of deep interplate earthquakes in Kanto, Japan presents a natural laboratory for illuminating the distribution of fault material heterogeneity. The region is situated in a special tectonic setting where the Philippine Sea plate (PHS) is “sandwiched” between the North American plate (NA) and Pacific plate (PAC) (*13*–*15*), generating abundant earthquakes at depths of 60 to 70 km along the plate interface between the PHS and PAC (Fig. 1). These deep interplate earthquakes are made possible by the relatively cold plate interface, estimated to have a temperature of ~300°C at depths of 60 to 70 km by thermal-mechanical models (*14*), allowing rocks to undergo brittle failure (*16*). If the PHS is not subducting between the NA and PHS, the plate interface could be hotter by nearly 300°C. Our study focuses on a region in eastern Kanto (35.4° to 36.4°N latitude and 139.5° to 140.5°E longitude). Compared to the shallower counterpart between the NA and PHS, the deeper plate interface between the PHS and PAC is more productive here, hosting magnitude (*M*) >3.5 earthquakes approximately every month that may be felt in the Tokyo metropolitan area.

**Fig. 2.**
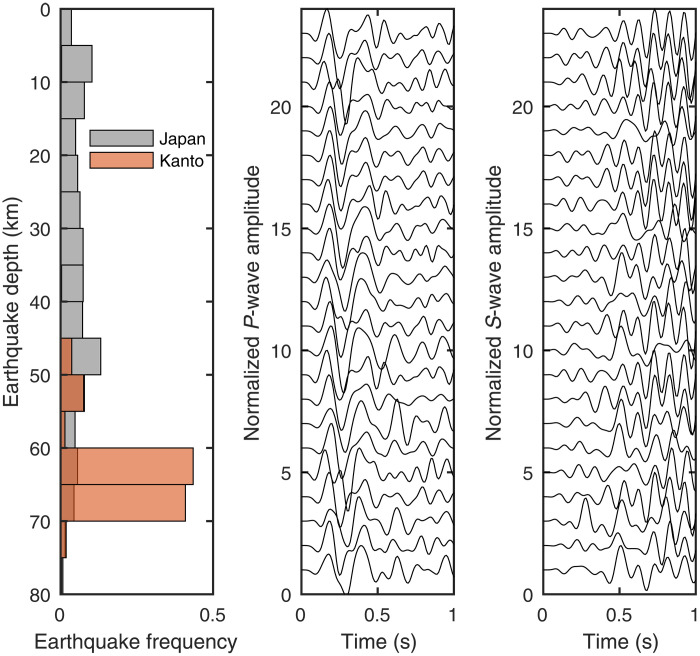
Earthquake frequency in the study region and *P* and *S* waveforms (2 to 10 Hz) for a cluster of similar earthquakes. The Japan and Kanto earthquakes in the histogram refer to *M* >2 earthquakes along the Japanese Islands and in the study region, respectively, recorded in the catalog of similar earthquakes (*17*).

The Kanto region is well instrumented by the high-sensitivity seismograph network (Hi-net) composed of borehole stations every ~25 km. The Hi-net stations provide an unprecedentedly rich dataset of more than 3000 *M*2 to *M*6 earthquakes at depths of 60 to 70 km between 2004 and 2020 (fig. S1 and Fig. 1). A close look at the data reveals highly similar waveforms for earthquakes close to each other (Fig. 2). Such similar earthquakes are especially abundant at depths of 60 to 70 km in the study region, whereas they mostly exist at shallower depths for the rest of Japan (Fig. 2) (*17*). We relocate the deep interplate earthquakes listed in the Japan Meteorological Agency (JMA) unified catalog using a double-difference algorithm (Materials and Methods) (*18*). The relocation results unveil localized patches of earthquake clusters (hereafter referred to simply as patches) distributed for tens of kilometers along strike at the same depth (Fig. 1). Earthquakes at 60- to 63-km depths are concentrated at a latitude of ~36°N, while earthquakes deeper than 64 km become more localized near the northern (36.1° to 36.2°N) and southern (35.6° to 35.7°N) ends (fig. S2). If we assume that each earthquake releases a stress drop of 3 MPa, a value consistent with the stress drop results shown later, the rupture areas of these earthquakes only account for ~7% of the study area, suggesting that most of the plate interface undergoes aseismic deformation at depths of 60 to 70 km.

## RESULTS

### Near-source *V*_p_/*V*_s_ ratio analysis

The localization of earthquakes suggests that their generation may be controlled by small-scale fault material heterogeneity. Previous tomography models imaged the spatial variation of seismic wave velocities and delineated the detailed slab geometry and slab contact zones (*19*–*21*). To illuminate fault material properties at scales relevant to earthquake sources, we take advantage of the Hi-net data of earthquake clusters to measure the in situ *V*_p_/*V*_s_ ratios, i.e., the ratio of *P*-wave velocity to *S*-wave velocity. The *V*_p_/*V*_s_ ratio is directly related to the Poisson’s ratio ν by $\nu =\frac{1}{2}\frac{{\left(V_{p}/V_{s}\right)}^{2}-2}{{\left(V_{p}/V_{s}\right)}^{2}-1}$. Typical rocks are considered to have an average *V*_p_/*V*_s_ ratio of 1.732, equivalent to a Poisson’s ratio of 0.25 (*22*). Tomography models found that the study area has typical or slightly higher *V*_p_/*V*_s_ ratios (1.7 to 1.9) at scales of tens of kilometers (*19*, *21*, *23*). *V*_p_/*V*_s_ ratio variations may be associated with mineral composition (*24*, *25*), fluid content (*26*, *27*), or cracks (*28*, *29*), as explained in more detail in the Discussion section. It was also shown that these deep interplate earthquakes occur immediately west of the downdip limit of a ~60-km wide low-velocity, high *V*_p_/*V*_s_ ratio region associated with the serpentinized mantle of the PHS (Fig. 1) (*21*).

**Fig. 3.**
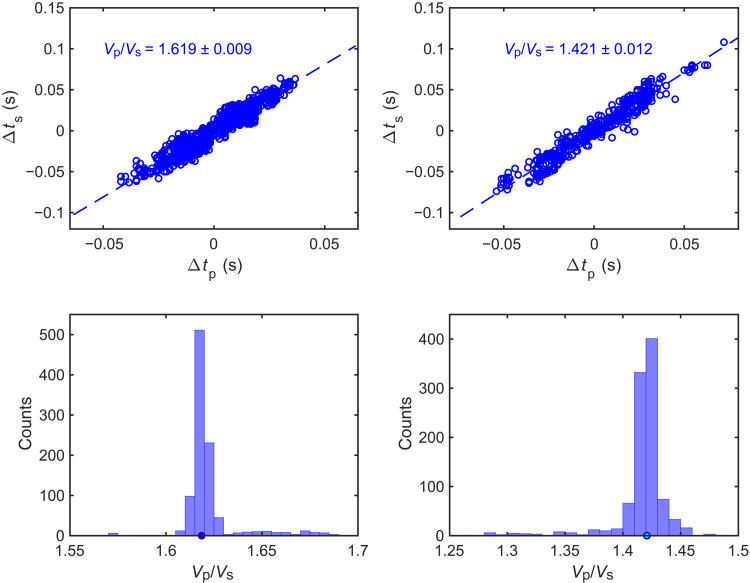
The demeaned differential *P*- and *S*-wave arrival times and the resulting *V*_p_/*V*_s_ ratios for two earthquake patches (top) as well as the distributions of *V*_p_/*V*_s_ ratios from the bootstrapping analysis (bottom).

We group *M*2 to *M*4 earthquakes at depths of 60- to 70-km to 1- to 2-km patches based on the relocation results (fig. S2). The earthquake patches are chosen to be spatially separated from each other and include 15 to 109 events. Given that the patch size is much smaller than the earthquake-station distance, the *V*_p_/*V*_s_ ratio can be inverted for each pair of events from the ratio of their differential *S*- and *P*-wave travel times, measured from horizontal (east and north) and vertical components, respectively. In practice, only the arrival times of seismic waves are recorded, and the *V*_p_/*V*_s_ ratios can be estimated using the *P*- and *S*-wave arrival times after they are demeaned by the average of arrival times from all the stations (Fig. 3), as proposed by Lin and Shearer (*30*). Hence, the resulting *V*_p_/*V*_s_ ratio represents the average *V*_p_/*V*_s_ ratio of the near-source region between earthquake pairs (~1 to 2 km), unlike the *V*_p_/*V*_s_ ratio in tomography models that is relevant to a much larger scale (>10 km). We visually inspect the Hi-net stations to select stations that record clear *P*- and *S*-wave arrivals for all the earthquakes in the cluster. We then filter the data using a fourth-pole Butterworth filter with a frequency band of 3 to 10 Hz, which is chosen to ensure enough event pairs with similar waveforms. We calculate the cross-correlation coefficients of event pairs and only use event pairs with cross-correlation coefficients higher than 0.85 for at least five stations. The differential arrival times are then demeaned and used to estimate the *V*_p_/*V*_s_ ratio using the robust linear regression method (*31*).

Compared to the previous implementation of the in situ *V*_p_/*V*_s_ ratio analysis (*30*), we incorporate new data processing steps to reduce the uncertainty in differential arrival time measurements and make the method more suitable for our dataset. We adopt two steps to choose the waveform window for cross-correlation to enhance the quality of cross-correlation results. First, we use the phase picks from the JMA catalog and handpick the arrivals for stations without JMA picks. To confirm the validity of arrival time picks, we invert the *P*- and *S*-wave arrival times for all the events in the same patch together, assuming that the arrival time *T_ij_* for each event *i* at a given station *j* contains an event term *ET_i_* and a station term *ST_j_*: *T_ij_ = ET_i_ + ST_j_*, where *ET_i_* should be the same for the *i*th event and *ST_j_* is the same for the *j*th station for *P* and *S* waves, respectively. We then verify that the inverted *P*- and *S*-wave arrival times at different stations for the same earthquake patch follow a constant slope (i.e., *V*_p_/*V*_s_ ratio along the wave propagation path). Second, we choose the waveform used for cross-correlation to start from the minimum arrival time for all the events at a given station and end at 1 s after the arrival time for each event at the same station (*T_1_*). Since we need to use the same window lengths for cross-correlation, we also apply zero padding to the waveforms after *T_1_*. The two steps can improve the accuracy in the estimation of differential arrival times considerably and make the *V*_p_/*V*_s_ ratios less dependent on the choice of cross-correlation coefficient thresholds and minimum number of stations.

Because this region has abundant repeating earthquakes (*17*), their differential arrival times may not be well resolved when they are below the resolution of the data sampling frequency. Thus, we also apply a distance threshold of 150 m to earthquake pairs to eliminate the effects of repeating earthquakes. This distance threshold is chosen as repeating earthquakes tend to be within 100 m from each other. Choosing a distance threshold of 200 m would reduce the number of event pairs with slight changes in *V*_p_/*V*_s_ ratio results. We also carry out a bootstrapping analysis to investigate further the uncertainty in *V*_p_/*V*_s_ ratio results due to differential arrival time measurements. We randomly resample the differential arrival times for event pairs 1000 times and calculate the *V*_p_/*V*_s_ ratios from the resampled datasets (Fig. 3).

This method of estimating in situ *V*_p_/*V*_s_ ratios, although powerful, may introduce bias to the results because of the differences between *P*- and *S*-wave ray paths in a heterogeneous medium (*32*). However, the amount of bias depends on the layout of stations and locations of earthquakes. To investigate the possible bias, we carry out synthetic tests by using the locations of an earthquake patch and the associated Hi-net stations (Materials and Methods and fig. S3). Our method can retrieve the almost exact *V*_p_/*V*_s_ ratio for a subduction zone model, even when earthquakes are surrounded by a local *V*_p_/*V*_s_ ratio anomaly (fig. S4). We also confirm that *V*_p_/*V*_s_ ratios can be underestimated or overestimated depending on the local *V*_p_/*V*_s_ ratio gradient in layered subduction zone models (*32*). The largest bias is introduced when we use a heterogeneous velocity model (*23*) with large model roughness and a wide range of *V*_p_/*V*_s_ ratios (1.5 to 2.0). The resulting *V*_p_/*V*_s_ ratio can be ~0.15 lower than the true value in this case, but adding a local *V*_p_/*V*_s_ ratio anomaly can counterintuitively reduce the bias (the fourth row of fig. S4). Therefore, it is reasonable to assume that our *V*_p_/*V*_s_ ratio results may have a bias of ~0.1 due to the limitation of the method.

**Fig. 4.**
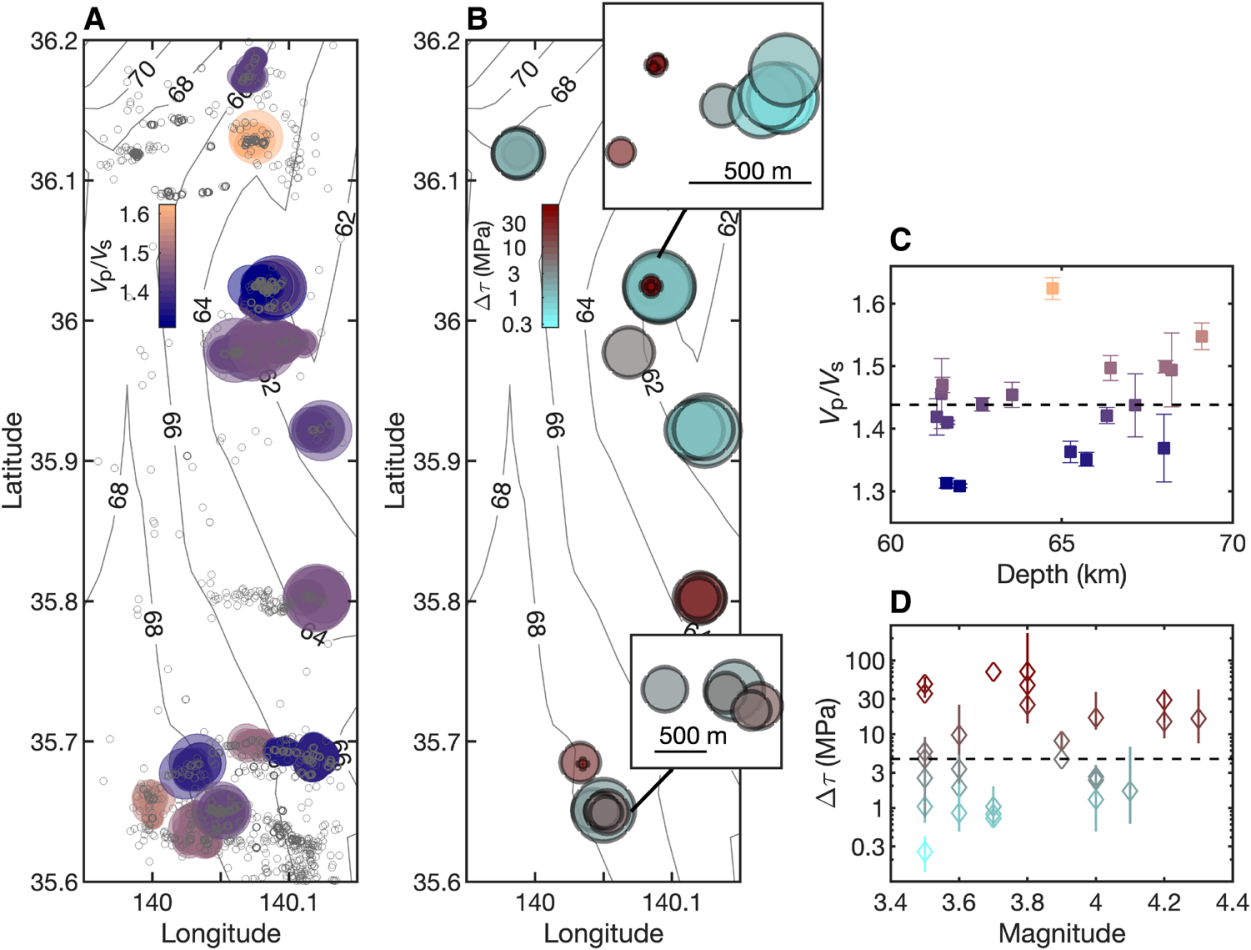
*V*_p_/*V*_s_ ratios of earthquake patches and stress drop of *M*3.5 to *M*4.3 earthquakes in the patches. (**A**) shows the locations of earthquakes colored by the *V*_p_/*V*_s_ ratios. The circle size is scaled by the rupture radius calculated from a stress drop of 3 MPa. The gray dots show the locations of *M*2 to *M*4 earthquakes. Depth contours of the PAC are obtained through linear interpolation of relocated earthquake depths and denoted by gray solid lines with numbers. (**B**) demonstrates the variations of stress drop. The circle size corresponds to eight times the rupture radius. The inset figures show the separation of high-stress-drop and low-stress-drop earthquakes on a real scale. (**C**) and (**D**) show the *V*_p_/*V*_s_ ratios of earthquake patches at different depths and the stress drops of *M*3.5 to *M*4.3 earthquakes, respectively, with the dashed lines showing the median values and the same marker color corresponding to earthquakes in the same patch. The error bars in *V*_p_/*V*_s_ ratios represent the median absolute deviation of the bootstrapping distribution, and the error bars in stress drop values represent the stress drops estimated from different eGfs.

We calculate the *V*_p_/*V*_s_ ratios of 18 Kanto earthquake patches by taking the average of the measurements from the east and north components of seismic data (Fig. 4, A and C, and table S1). The differences in *V*_p_/*V*_s_ ratios between the two components are below 0.12 except for two patches. Furthermore, the median *V*_p_/*V*_s_ ratio measured from the east component is almost identical to that from the north component (fig. S5). The resulting *V*_p_/*V*_s_ ratios are anomalously low with a median of 1.44, equivalent to a Poisson’s ratio of nearly 0. Because most *V*_p_/*V*_s_ ratio measurements are within 0.1 from the median, the observed range of measurements is at least partially caused by the method bias discussed above. Except for the cluster with the highest *V*_p_/*V*_s_ ratio, the results are spatially stable (Fig. 4A) for different patches that are measured by a different subset of Hi-net stations depending on the data availability. We also notice that some earthquake patches are more elongated in the north-south or west-east direction (fig. S2), but their shape has a minimal effect on the resulting *V*_p_/*V*_s_ ratios.

### Stress drop analysis

To further understand fault stress conditions that host these interplate earthquakes, we measure the stress drops of *M*3.5 to *M*4.3 earthquakes using a spectral ratio approach (*33*, *34*) based on the assumption that smaller nearby earthquakes, i.e., empirical Green’s functions (eGfs), can be used to cancel out the wave propagation and site effects and thus obtain the source parameters of target earthquakes. We require the eGfs to be at least one magnitude smaller and located within one source dimension from the target events that are initially assumed to have a stress drop of 1 MPa. We measure the spectral ratios using five overlapping *P*-wave windows and averaged over the Hi-net stations (Materials and Methods). Because each target event can have multiple eGfs due to the high seismicity level within one cluster, we report the average stress drop and the range of stress drop estimates from different eGfs for the same target earthquake (Fig. 4, B and D). The resulting stress drop has a range of 0.3 to 70.1 MPa with a median of 4.6 MPa, which is comparable to that of shallow crustal earthquakes observed using the same method (*34*). The typical stress drop values suggest that the effective normal stress on faults is likely much lower than the pressure at 60 to 70 km (~1.5 GPa), indicating that the fault zone may contain abundant fluid (*35*, *36*), although stress drops can be invariant to normal stress if earthquake ruptures are confined (*37*). We also note that earthquakes with similar stress drops, whether they are high or low, are located close to each other (Fig. 4B), suggesting that the amount of stress that can be released in each earthquake is related to its location on the plate interface.

## DISCUSSION

### Implications for material heterogeneity

**Fig. 5.**
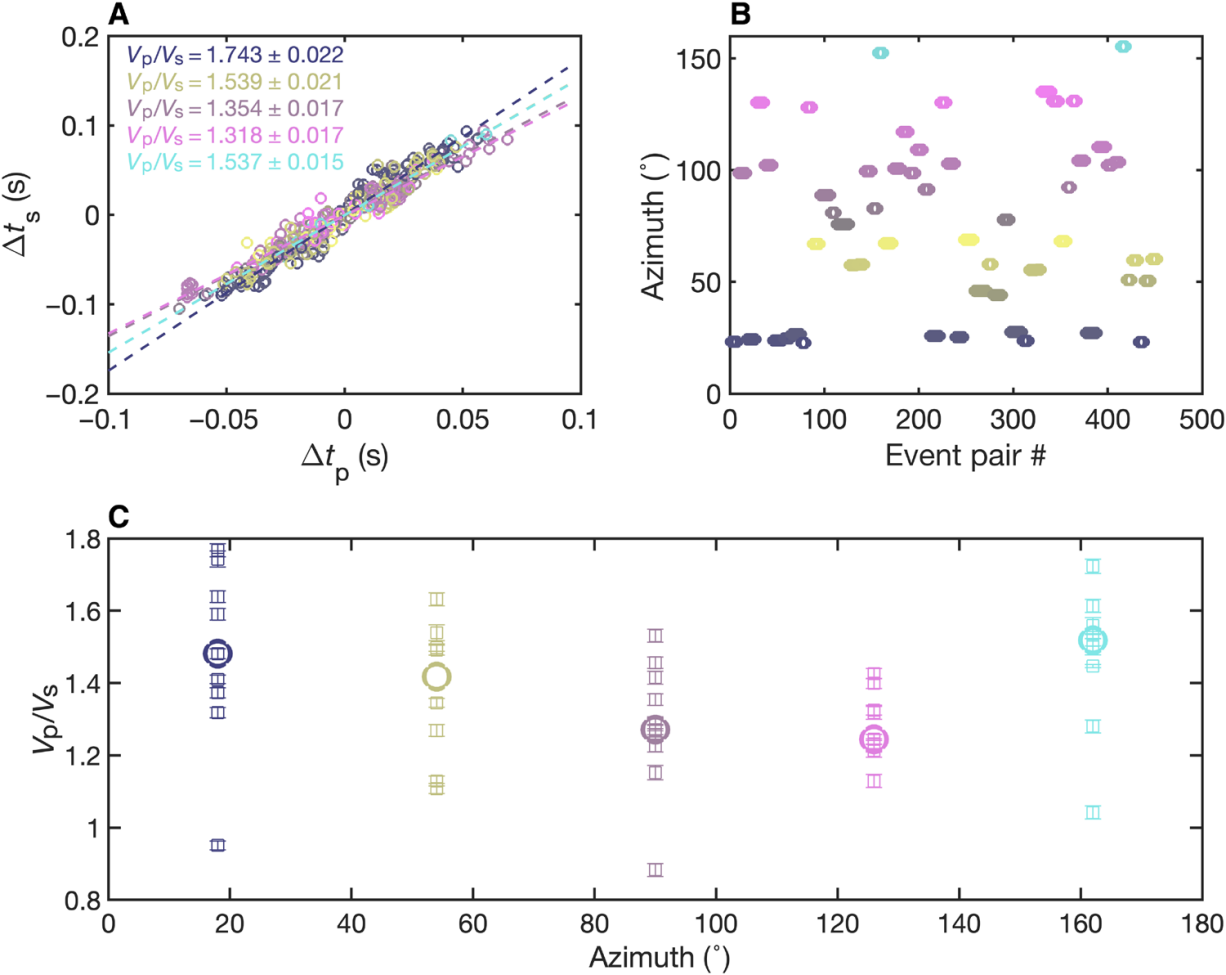
The azimuthal dependence in *V*_p_/*V*_s_ ratios. (**A**) and (**B**) demonstrate the calculation of *V*_p_/*V*_s_ ratios using earthquake pairs in five azimuthal zones for one earthquake patch and the associated azimuths of event pairs. (**C**) shows the azimuthal dependence for earthquake patches with more than 200 event pairs. The circle denotes the median value for each zone.

It has been shown that anisotropic structures can cause very low *V*_p_/*V*_s_ ratios in certain directions (*29*, *38*, *39*). Because we measure differential travel times by linking earthquake pairs located in various directions with respect to each other, the results represent the *V*_p_/*V*_s_ ratios averaged from different event pair orientations. Hence, we investigate whether there is strong azimuthal dependence of the *V*_p_/*V*_s_ ratios in the earthquake source region. We divide the azimuths of event pairs, which have a range of 0° to 180° from north, to five equal zones and calculate the *V*_p_/*V*_s_ ratios using event pairs within each zone (Fig. 5, A and B). Most of the patches show higher *V*_p_/*V*_s_ ratios close to the north-south direction and lower *V*_p_/*V*_s_ ratios near the west-east direction (Fig. 5C) with an averaged anisotropy amplitude of 14.5% (fig. S6). Since each *V*_p_/*V*_s_ ratio takes account of arrival time measurements from stations over various propagation path directions, the strong azimuthal dependence of the resulting *V*_p_/*V*_s_ ratios is more likely to reflect the anisotropic structure in a small volume in the near-source region (fig. S7). The similar azimuthal-dependence of *V*_p_/*V*_s_ ratios for different earthquake patches also suggests that the near-source anisotropic structure may be persistent along the strike of the subduction zone.

Such azimuthal dependence of *V*_p_/*V*_s_ ratios can be caused by a highly damaged shear zone with foliated rocks (*40*). Assuming transverse isotropy, the symmetry axis tends to be nearly horizontal and close to either the north-south or west-east direction given the large differences of *V*_p_/*V*_s_ ratios between the two directions. In other words, the foliation planes in the shear zone should be aligned close to the along-strike or along-dip directions on the Pacific slab. Such anisotropy structure alone, however, cannot fully explain the observed low *V*_p_/*V*_s_ ratios, as the median of the relatively high *V*_p_/*V*_s_ ratios in the north-south direction (~1.5) is still much lower than the regional-scale *V*_p_/*V*_s_ ratios resolved in tomography models, suggesting that other mechanisms may also contribute to the low *V*_p_/*V*_s_ ratios of the source medium.

Three possible mechanisms may account for the inherently low *V*_p_/*V*_s_ ratios of the near-fault region. One natural explanation is the existence of distinct mineral composition such as alpha quartz, which has a *V*_p_/*V*_s_ ratio of 1.48. Quartz veins in shear zones are parallel or subparallel to the foliation, potentially contributing to the observed anisotropic effect (*41*). Another possibility is that the source regions contain cracked rocks saturated with highly compressible fluid. Although more fluid is typically assumed to increase *V*_p_/*V*_s_ ratios, it is shown that the *V*_p_/*V*_s_ ratio can either increase or decrease with increasing fluid content, depending on the pore aspect ratio, fluid compressibility, and the initial Poisson’s ratio of the solid part (*27*, *28*). The equilibrium geometries of the rock and fluid system lead to intermediate aspect ratios (~0.15 to 0.5) (*28*), which, in combination with highly compressible fluid, allow the Poisson’s ratio to decrease with increasing porosity until a certain threshold (*27*). Lastly, although tomography models showed that the earthquakes occurred deeper than the serpentinized mantle (*21*), the slab interface may be overlain by a several-kilometer-thick serpentinized channel that cannot be resolved in tomography models (*42*). Such serpentinized channels are considered to be highly anisotropic with *V*_p_/*V*_s_ ratios ranging between 1.3 and 2.8 (*38*). These serpentinite-enriched shear zones can promote fluid overpressure through metasomatic reactions, which can produce deep episodic tremors in other subduction zones (*40*).

We also note that 6 of 18 patches have *V*_p_/*V*_s_ ratios lower than $\sqrt{2}$ or 1.414 but higher than 1.3, indicating negative Poisson’s ratios. This could be caused by the limitation of our method, which can introduce a bias of ~0.1 as discussed previously; however, it is also possible for natural minerals to have negative Poisson’s ratios. For example, the abovementioned serpentinite aggregate (antigorite and deformed serpentinites) can have very high seismic anisotropy and exhibit a *V*_p_/*V*_s_ ratio as low as 1.3, which is due to the remarkable ranges of *V*_p_ (5.8 to 8.3 km/s) and *V*_s_ (2.9 to 4.7 km/s) (*38*). For isotropic conditions, alpha-cristobalite can also have negative Poisson’s ratios at a wide range of temperature (20° to 1500°). Quartz-rich sedimentary rocks exhibit negative Poisson’s ratios at confining pressures lower than 200 MPa due to its main constituent mineral alpha quartz and the existence of microcracks and micropores (*43*).

We acknowledge that there may exist other mechanisms that can reduce *V*_p_/*V*_s_ ratios, but they likely support the existence of a heterogeneous near-fault medium with distinct material properties from those of surrounding rocks. The observation of anisotropic *V*_p_/*V*_s_ ratios supports the existence of a damaged and foliated medium, and the observation of typical stress drop values suggest low effective normal stress and thus high fluid pressure is a plausible contributor to the generation of earthquakes. Such fault material heterogeneity is highly localized, leading to localized earthquake patches along the plate interface. The damaged and foliated medium also facilitates fluid migration and elevates fluid pressure, which can lower the effective normal stress and thus frictional strength on faults, enabling the frequent occurrence of these deep interplate earthquakes. Note that because *V*_p_/*V*_s_ ratios are measured from differential arrival times of earthquakes, their values represent the physical state of the fault zone during earthquakes. Thus, such damaged and foliated medium may heal during the interseismic period, allowing the cyclic changes in fluid pressure and the so-called fault valving behavior (*12*).

Small-scale fault zone heterogeneities have also been proposed to promote slow-slip behaviors. For example, a combination of drilling and seismic data analysis revealed fault zone heterogeneity over length scales from centimeters to kilometers in the source regions of slow-slip events at the Hikurangi subduction zone (*44*). Whether the fault zone slips seismically or aseismically likely depends on the rheological and mechanical behaviors of rocks inside. Numerical models of fault zones containing a mixture of frictional and viscous materials can reproduce the broad spectrum of seismic and aseismic slip behaviors (*45*–*48*). Hence, depending on the rheological and mechanical behaviors of fault zones, small-scale material heterogeneity observed in this study may promote aseismic slip transients in other subduction zones with different temperature and pressure conditions. Identifying such small-scale fault material heterogeneity is an important first step for advancing our understanding of the patchy behaviors or localization of seismic and aseismic slip (*44*).

**Fig. 6.**
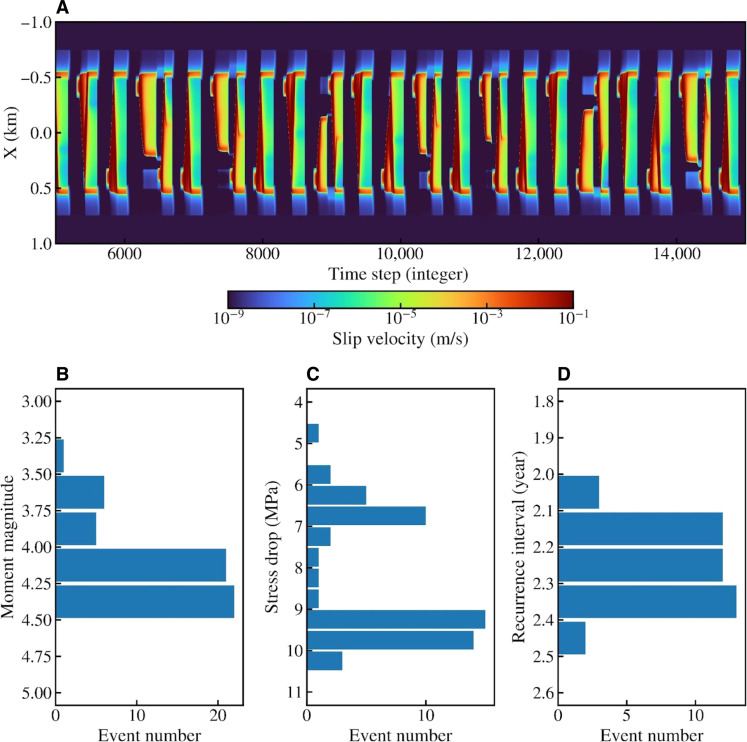
Results from earthquake cycle simulation of *M* ~ 4 earthquakes with model parameters shown in table S2. (**A**) shows the temporal evolution of fault slip rate with orange and red colors corresponding to earthquake rupture and the rest associated with interseismic and postseismic periods. (**B** to **D**) demonstrate the moment magnitude, stress drop, and recurrence intervals of modeled earthquakes.

To further test the hypothesis that the damaged and foliated fault zone enables fluid propagation and weakens the plate interface, we compare the recurrence intervals of *M*4 Kanto earthquakes with those of simulated *M*4 earthquakes in fully dynamic earthquake cycle simulations (Materials and Methods). On average, *M*4 Kanto earthquakes at the same location in the study region repeat every 2 to 5 years, which can be reproduced in our simulations if an effective normal stress of tens of megapascals is assumed (Fig. 6). Such low effective normal stress requires elevated fluid pressure in the fault zone at the deep plate boundary, as the lithostatic confining pressure at this depth range is ~1.5 GPa. Moreover, the recurrence intervals of simulated *M*4 earthquakes are less dependent on frictional parameters than the effective normal stress. Simulations with different frictional parameters have qualitatively similar results as long as the effective normal stress is kept at tens of megapascals. For example, small characteristic slip distance leads to more variability in earthquake magnitude and stress drop (Fig. 6), but it does not substantially change the recurrence intervals of simulated *M*4 earthquakes. However, using an effective normal stress of several hundred megapascals would lead to much higher stress drops and longer recurrence intervals of *M*4 earthquakes. Hence, the simulation results are consistent with the notion that a fluid-enriched fault zone facilitates the repeating ruptures of these deep interplate earthquakes.

Our observation has its own limitations as the method only provides the *V*_p_/*V*_s_ ratio measurements rather than independent constraints on *V*_p_ or *V*_s_. Having independent velocity or density measurements can help narrow down the possible environment hosting the deep interplate earthquakes, but, currently, it is still challenging to measure these elastic properties in localized earthquake patches. In conclusion, our results provide the first evidence of localized anisotropic fault structures with anomalously low *V*_p_/*V*_s_ ratios, corresponding to a nearly zero Poisson’s ratio, at the scale relevant to earthquake sources (~1 to 2 km). These structures, which have not been identified using conventional macroscopic investigation methods such as seismic tomography and receiver functions, should be related to microscopic geological observation of foliation, cracks, and channels and may contain multifault networks with meters-thick faults (*49*). Such small-scale material heterogeneity may cause stress perturbations on faults that in turn form an ideal environment for the occurrence of earthquakes. The size and spatial distribution of these structures would control the maximum size of earthquakes, and a further understanding will contribute to the assessment of seismic hazards in the Japan subduction zone.

## MATERIALS AND METHODS

### Earthquake relocation

We relocate earthquake hypocenters in the Kanto region from 1 January 2003 to March 2022, using the double-difference relocation method (*18*). We use 9668 *M* ≥ 2 earthquakes listed in the JMA unified catalog in the analysis. Among them, 3360 *M* ≥ 2 events are located near the upper boundary of the PAC, at a depth of 60 to 70 km. Our relocation process follows Shelly *et al.* (*50*) and Yoshida and Hasegawa (*51*) assuming the one-dimensional (1D) velocity model (*52*) used in the JMA catalog. We use the differential arrival times of 715,228 *P*-wave and 629,505 *S*-wave data from the JMA unified catalog. In addition, we use 4.9 million *P*-wave and 2.7 million *S*-wave data obtained from waveform correlation analysis from 28 local and regional stations of the National Institute for Earth Science and Disaster Prevention (NIED) Hi-net (NIED, 2019), national universities, and the JMA. *P*- and *S*-wave windows of 2.5 and 4.0 s, respectively, are used for correlation analysis, starting 0.3 s before onset. We use manually picked arrival time data listed in the JMA unified catalog if available; otherwise, we use theoretical arrival times computed by assuming the same velocity model as used in the JMA unified catalog. We calculate cross-correlation functions for all event pairs with horizontal distances of less than 10 km by applying two band-pass filters, one at 2 to 5 Hz and the other at 5 to 12 Hz, for all available components. We adopt the lag times obtained for the component and frequency band with the highest correlation coefficient for the same station of the same pair. After the relocation process, the mean residual of the differential arrival time data from waveform correlation is reduced from 58 to 21 ms.

### Synthetic tests of *V*_p_/*V*_s_ ratios

To investigate the effects of different P and S ray paths on the resulting *V*_p_/*V*_s_ ratio estimates, we conduct synthetic tests using subduction models of different velocity structures and *V*_p_/*V*_s_ gradients. *P* and *S* waves propagate through the models from one of the earthquake clusters used in the real data analysis, resulting in *P*- and *S*-wave travel times at the corresponding receivers. We simulate the travel time wavefield using a fast-marching method as implemented in the open-source code PyKonal (*53*), which has been demonstrated to have high numerical accuracy. The wavefield is evaluated on a 1 km–by–1–km–by–1 km Cartesian grid augmented with five times denser sampling near the virtual source and receiver. The synthetic travel time data are then used for *V*_p_/*V*_s_ ratio analysis as if the real data.

We consider four velocity models with different velocity complexities and *V*_p_/*V*_s_ gradients, including (i) a subduction zone model based on the AK135 (*54*) with one east-dipping subducting slab above the earthquake cluster, (ii) a layered model with two west-dipping slabs mimicking the sandwich model and the earthquake cluster located within the lower slab, (iii) a layered model for the top 55 km underlain by a 3D tomography model (*23*), and (iv) a pure 3D tomography model (*23*). Figures S3 and S4 demonstrate the model setups as well as the true and recovered *V*_p_/*V*_s_ ratios for each model. Note that the 3D tomography model contains large roughness, and, in the travel time calculations, we constrain the *V*_p_/*V*_s_ ratios to fall between 1.5 and 2.0 by adjusting any out-of-range values to the nearest end values. For model (iii), we further add a random Gaussian noise with SDs of 0.02 and 0.032 s to each *P*- and *S*-wave travel time calculation, respectively. Although the distributions of differential travel times in this case are more spread out than those in the real data, we find that adding noise only slightly changes the estimated *V*_p_/*V*_s_ ratio (third row of fig. S4), likely because the travel time perturbations caused by the velocity structure heterogeneity are comparable to the noise level.

### Stress drop analysis

After identifying eGfs that are at least one magnitude smaller, we calculate the spectral ratios between the target earthquakes and their eGfs using *P* waves recorded on the vertical component. The *P*-wave window starts 0.05 s before the direct *P*-wave arrival time used in the *V*_p_/*V*_s_ ratio analysis. We taper the *P*-wave windows using a 5% Tukey window and apply five windows overlapped by half of the window duration to stabilize the spectral ratios (*34*). We also sample the spectrum at equal intervals of 0.025 in log frequency and smooth it with a running average for every five samples. We then fit the spectral ratio using trust region reflective optimization (*55*), assuming that the earthquakes observe the typically assumed Brune spectral model (*56*). To minimize the influence of window lengths on spectral estimates, we search the window length that gives the smallest misfits between the Brune spectral model and the stacked spectral ratios over available stations. We convert the resulting corner frequency to stress drop estimates assuming a circular crack model (*57*) and Sato and Hirasawa’s source model parameter (*58*), which lead to intermediate stress drop values among other source models. The local *P*-wave velocity is assumed to be 8 km/s based on the *P*-wave velocities at 60 to 70 km in the preliminary reference Earth model (*59*).

### Fully dynamic earthquake cycle models

We simulate *M* ~4 earthquakes with a 2D antiplane model where the displacement is out of the plane of interest, which corresponds to an along-strike cross section of the plate interface. The fault is a 2-km-long line in an elastic medium, and the parallel lateral boundary is 1.6 km away. In addition to the dynamic fault, all other boundaries are absorbing boundaries. Rate and state dependent friction (“aging” law) is applied to control the fault slip behavior (*60*, *61*)

$$\frac{\tau }{\sigma_{n}}=\mu^{*}+\mathrm{aln}\left(\frac{V}{V^{*}}\right)+\mathrm{bln}\left(\frac{V^{*}\theta }{D_{\mathrm{RS}}}\right)$$ (1)

where τ is the frictional strength, σ_*n*_ is the normal stress, *V* is the sliding velocity, θ is the state variable indicating the real area of contact, and μ* and V* are reference values of friction coefficient and slip velocity. The earthquake asperity (velocity weakening) is in the center of the fault with a width of 1 km, and two strong barriers (velocity strengthening) are set up to hinder the outward penetration of coseismic rupture. The distributions of *a* and *b* are symmetric with respect to the middle of the fault. All key parameters are summarized in table S2. We use a spectral element method to simulate earthquake cycles with full inertial effects (*62*) and ensure numerical convergence by including at least five grid points in the process zone (~30 m).
