# Supplementary material for: Fault material heterogeneity controls deep interplate earthquakes
Source: Sci Adv. 2025 Feb 26;11(9):eadr9353. doi: 10.1126/sciadv.adr9353 (PMC11864176; doi:10.1126/sciadv.adr9353)
Supplement: Supplementary file 1 — Figs. S1 to S7 Tables S1 and S2 [file sciadv.adr9353_sm.pdf]

Science Advances
AAAS

# Supplementary Materials for

## Fault material heterogeneity controls deep interplate earthquakes

Yihe Huang *et al.*

Corresponding author: Yihe Huang, yiheh@umich.edu

*Sci. Adv.* **11**, eadr9353 (2025)
DOI: 10.1126/sciadv.adr9353

**This PDF file includes:**

Figs. S1 to S7
Tables S1 and S2

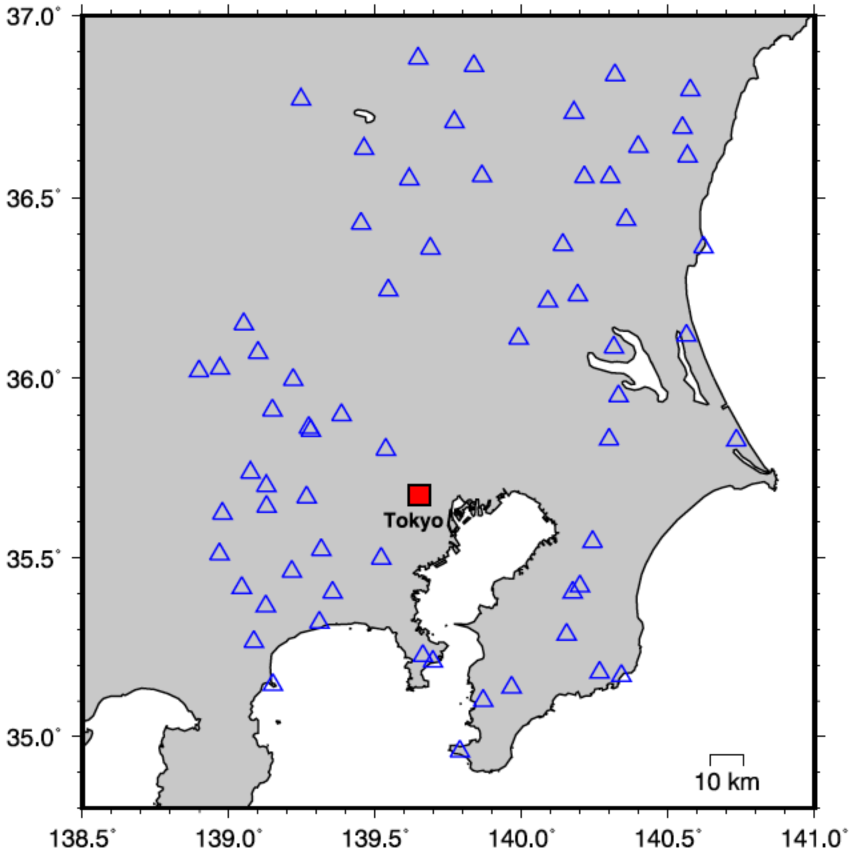


**Fig. S1. Spatial distribution of Hi-net stations used in this study.**

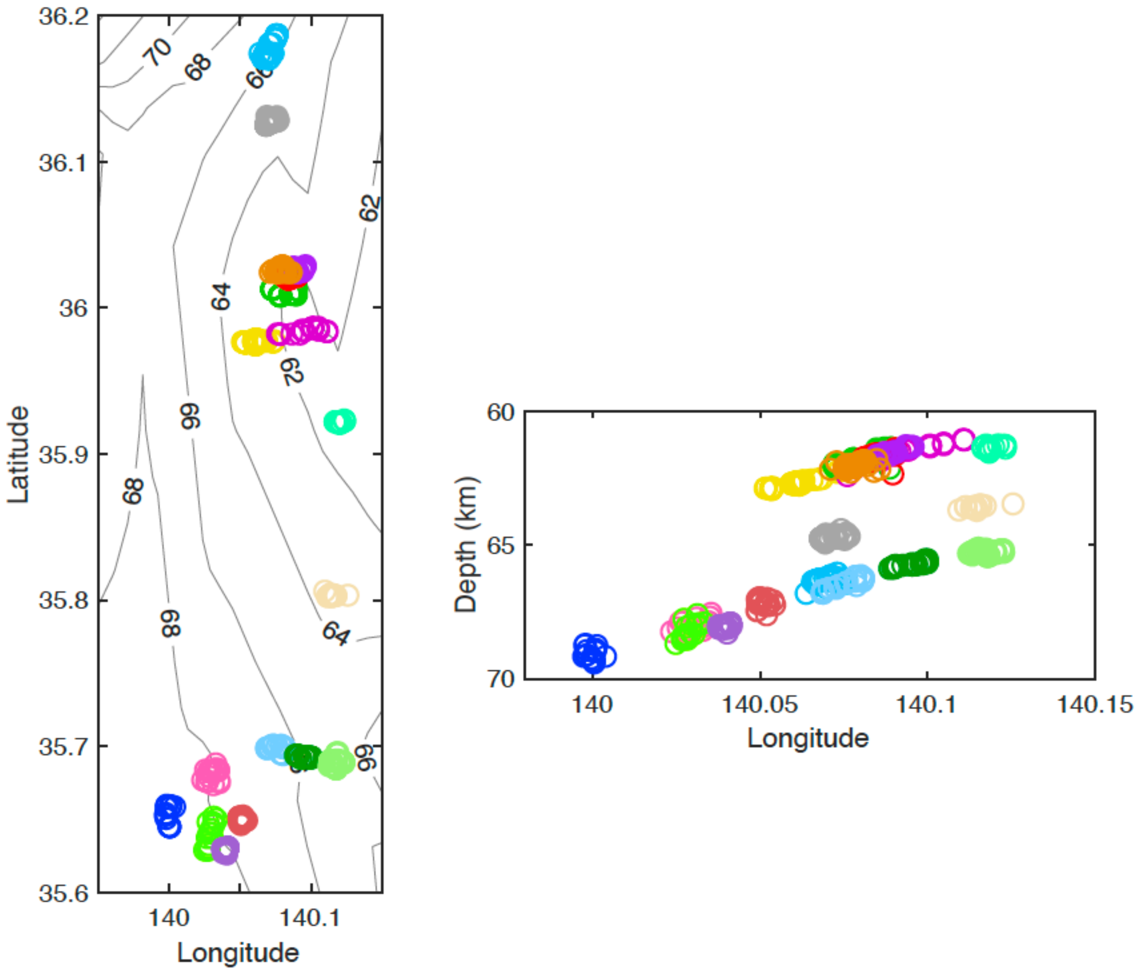


**Fig. S2. The map view and depth distribution of 18 earthquake patches used in the analysis.** Depth contours of the Pacific plate are obtained through linear interpolation of relocated earthquake depths based on the MatLab scatteredInterpolant function and denoted by gray solid lines with numbers.

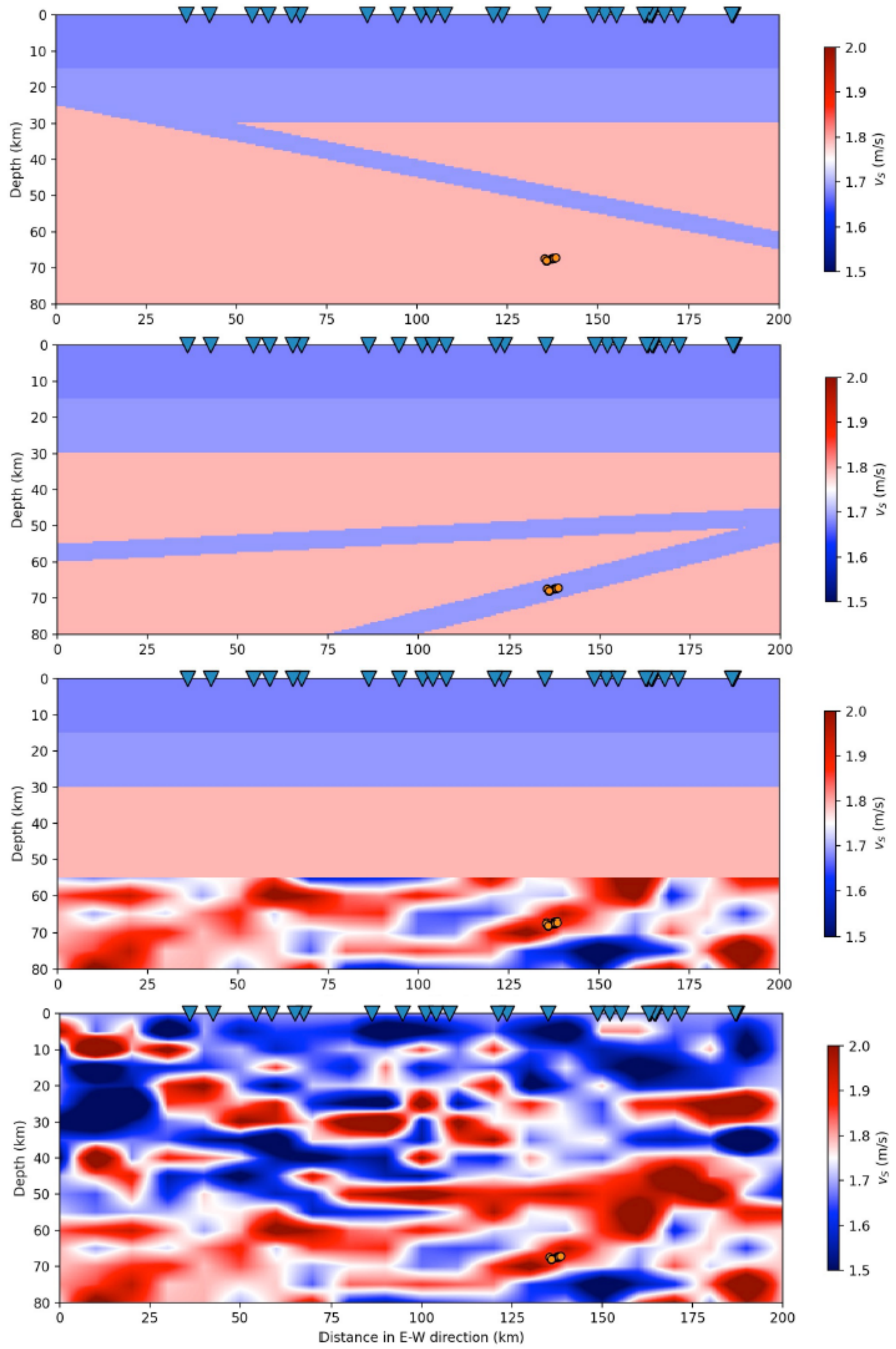


**Fig. S3. The four velocity models used in the synthetic tests shown in fig. S4.** From top to bottom: I) a subduction zone model with one east-dipping subducting slab above the earthquake cluster (circles); II) a layered model with two west-dipping slabs with the earthquake cluster located within the lower slab; III) a layered model for the top 55 km underlain by a 3D tomography model; IV) a pure 3D tomography model.

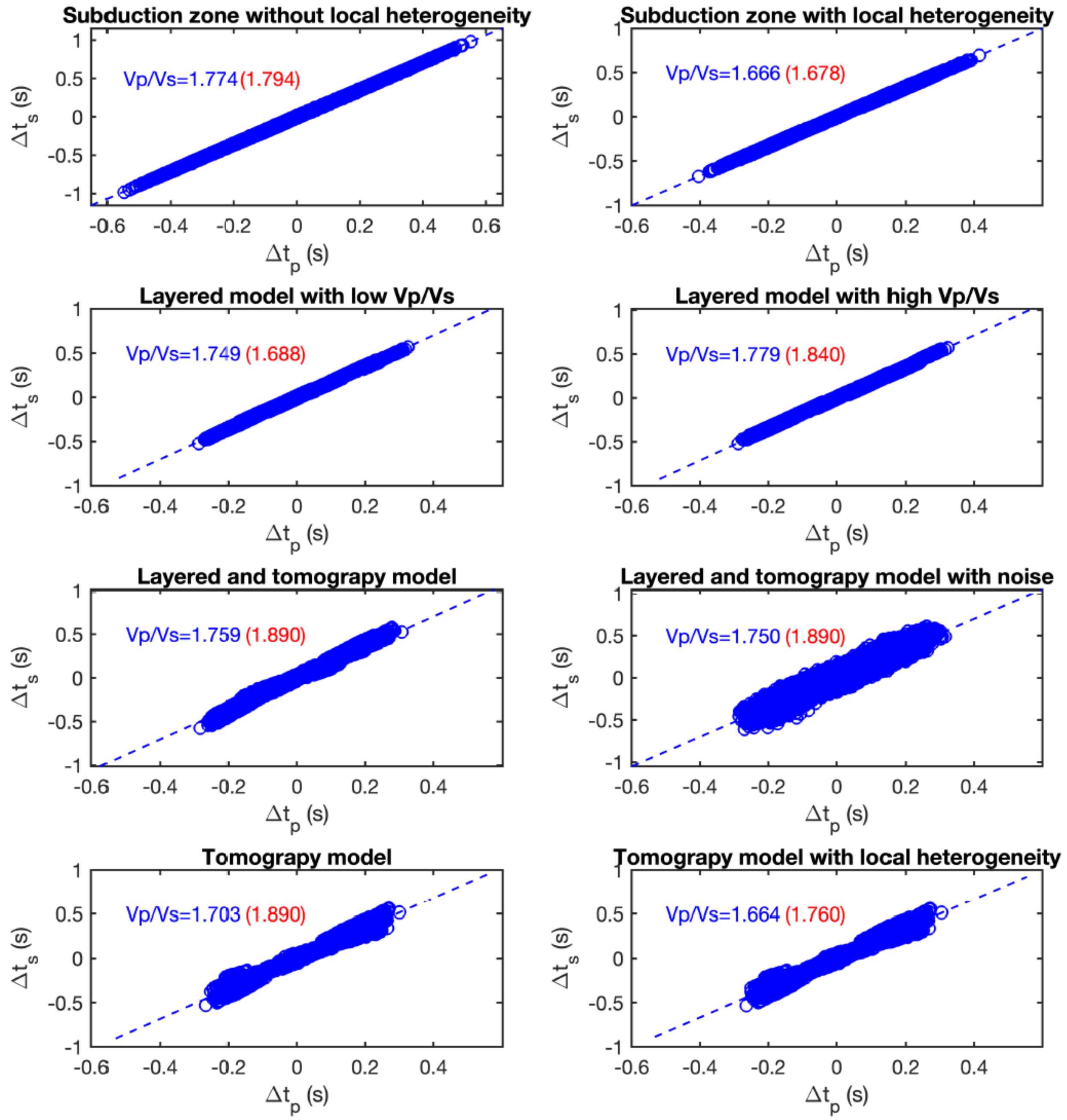


**Fig. S4. Vp/Vs ratios estimated from the synthetic tests (blue) along with the true values (red).** Please refer to fig. S3 for the corresponding model setups for each row. For models with local heterogeneity, the earthquake cluster is surrounded by a rectangular region with a different Vp/Vs ratio.

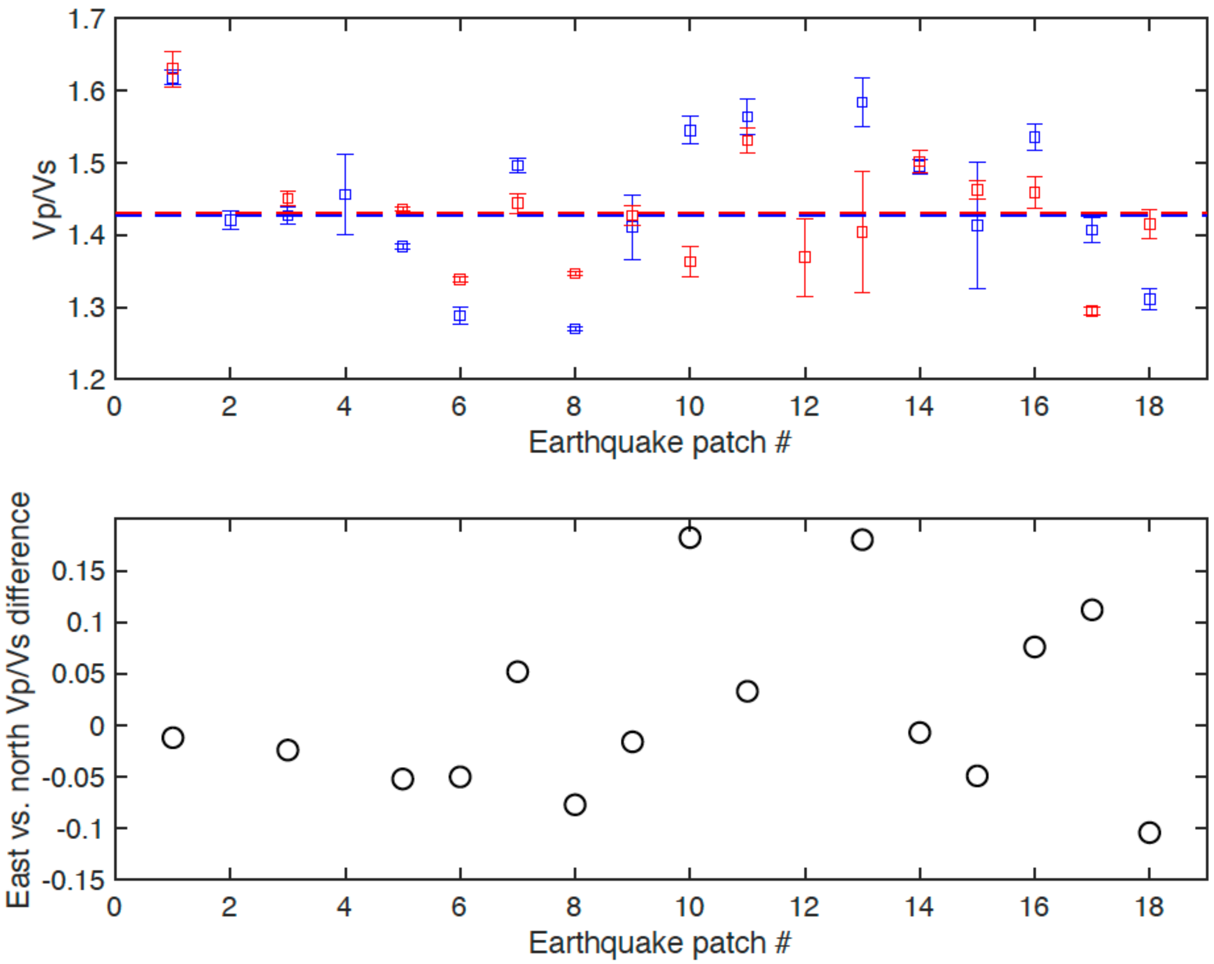


**Fig. S5. Vp/Vs ratios estimated from east (blue) and north (red) components (top) as well as the differences between Vp/Vs ratios estimated from east and north components when the patch has both measurements (bottom).** The dashed lines show the median Vp/Vs ratios for the two components.

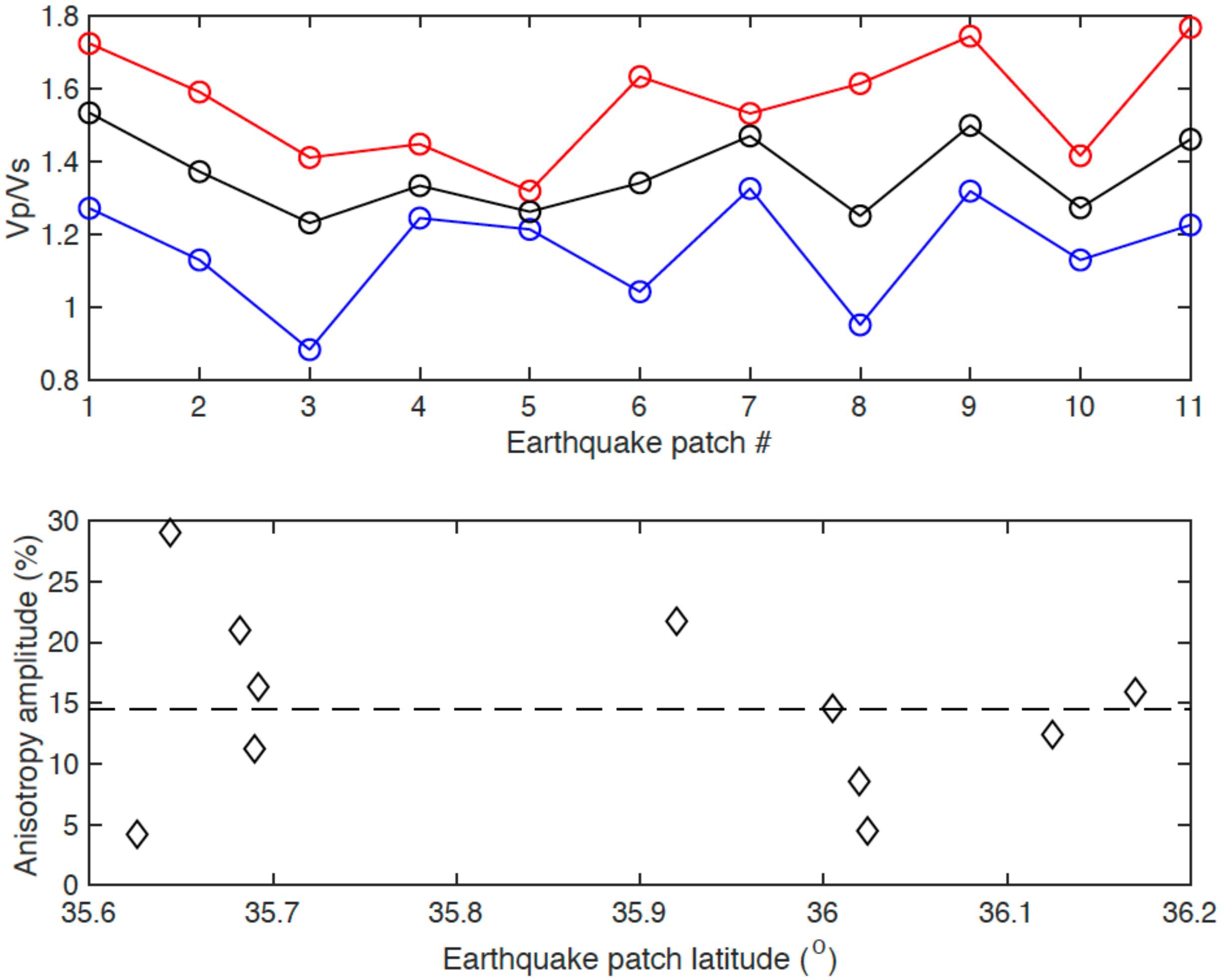


**Fig. S6. Maximum (red), mean (black), and minimum (blue) Vp/Vs ratios estimated using earthquake pairs in 5 azimuthal zones for each earthquake patch with more than 200 event pairs (top) as well as the anisotropy amplitude as a function of earthquake patch latitude (bottom).** The dashed line shows the mean amplitude. The anisotropy amplitudes are calculated by dividing the differences between maximum and mean Vp/Vs ratios by the mean values.

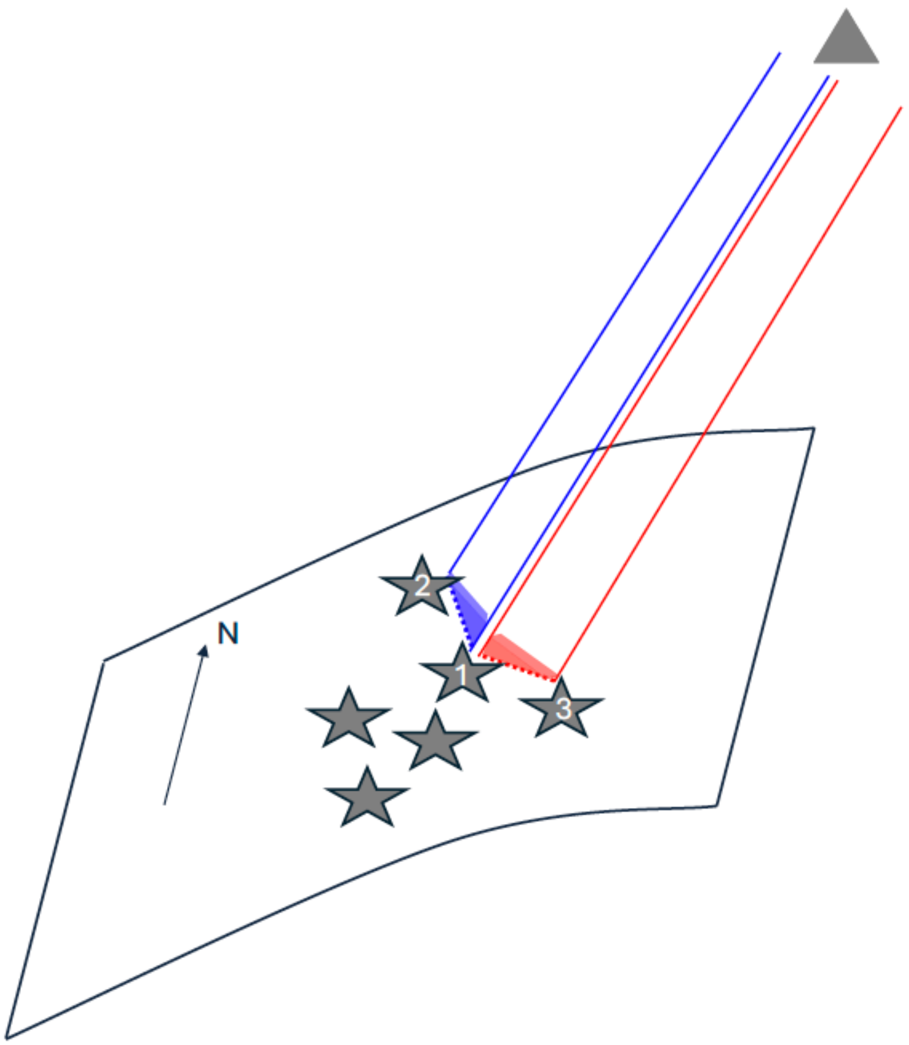


**Fig. S7. The ray path propagation directions from events #1, #2 and #3 to a distant station.** The blue and red regions denote the two different near-source volumes for which Vp/Vs ratios are measured when using events #1 and #2 as well as events #1 and #3, respectively.

**Table S1. Vp/Vs ratios of Kanto earthquake patches**

| Earthquake patch longitude range (ºE) | Earthquake patch latitude range (ºN) | Depth range (km) | Vp/Vs ratio |
|---|---|---|---|
| 140.067–140.077 | 36.124–36.131 | 64.4–65.0 | 1.624±0.018 |
| 140.063–140.076 | 36.170–36.188 | 66.0–66.8 | 1.421±0.013 |
| 140.050–140.074 | 35.974–35.979 | 62.2–63.0 | 1.439±0.011 |
| 140.075–140.111 | 35.981–35.987 | 61.3–62.5 | 1.456±0.056 |
| 140.072–140.092 | 36.008–36.014 | 61.0–62.2 | 1.410±0.003 |
| 140.08–140.091 | 36.019–36.024 | 61.3–62.5 | 1.313±0.008 |
| 140.084–140.096 | 36.024–36.029 | 61.2–61.7 | 1.470±0.012 |
| 140.070–140.086 | 36.024–36.029 | 61.7–62.3 | 1.309±0.003 |
| 140.116–140.124 | 35.920–35.924 | 61.2–61.6 | 1.419±0.029 |
| 140.109–140.126 | 35.802–35.807 | 63.4–63.8 | 1.454±0.020 |
| 139.997– 140.004 | 35.644–35.660 | 68.7–69.5 | 1.548±0.022 |
| 140.023– 140.036 | 35.673–35.689 | 67.5–68.3 | 1.369±0.054 |
| 140.023– 140.034 | 35.628–35.652 | 67.5–68.7 | 1.494±0.059 |
| 140.037– 140.042 | 35.626–35.632 | 67.9–68.3 | 1.499±0.010 |
| 140.049– 140.055 | 35.646–35.653 | 66.9–67.6 | 1.438±0.051 |
| 140.068– 140.081 | 35.694–35.702 | 66.1–66.8 | 1.497±0.020 |
| 140.088– 140.100 | 35.691–35.695 | 65.5–65.9 | 1.351±0.011 |
| 140.112–140.123 | 35.684–35.696 | 65.1–65.5 | 1.363±0.017 |

The error bars in Vp/Vs ratios represent the median absolute deviation of the bootstrapping distribution.

**Table S2. Earthquake cycle model parameters**

| **Frictional properties (within asperity)** | Symbol | Value |
|---|---|---|
| Reference friction coefficient | $\mu^*$ | 0.6 |
| Reference slip velocity | $V^*$ | $10^{-6}$ m/s |
| Plate loading rate | $V_{pl}$ | $10^{-9}$ m/s |
| Direct effect (constant) | $a$ | 0.006 |
| Ratio of a to b | $a/b$ | 0.5 |
| Characteristic slip distance | $D_{RS}$ | 0.4 mm |
| **Physical properties** | | |
| Effective normal stress | $\sigma_n$ | 50 MPa |
| Shear wave speed | $C_s$ | 4480 m/s |
| Shear modulus of host rock | $G$ | 53.2 GPa |
| Asperity size | $W$ | 1 km |
